# Cell Wall *N*-Linked Mannoprotein Biosynthesis Requires Goa1p, a Putative Regulator of Mitochondrial Complex I in *Candida albicans*

**DOI:** 10.1371/journal.pone.0147175

**Published:** 2016-01-25

**Authors:** Xiaodong She, Richard Calderone, Michael Kruppa, Douglas Lowman, David Williams, Lili Zhang, Ying Gao, Kasra Khamooshi, Weida Liu, Dongmei Li

**Affiliations:** 1 Georgetown University Medical Center, Department of Microbiology & Immunology, Washington, DC, 20057, United States of America; 2 Institute of Dermatology, Chinese Academy of Medical Sciences (CAMS), Jiangsu Key laboratory of Molecular Biology for Skin Disease and STIs, Nanjing, 210029, China; 3 Department of Surgery, Department of Biomedical Sciences and Center of Excellence in Inflammation, Infectious Diseases, and Immunity, East Tennessee State University, Johnson City, Tennessee, 37614, United States of America; New Jersey Medical School, Rutgers University, UNITED STATES

## Abstract

The Goa1p of *Candida albicans* regulates mitochondrial Complex I (CI) activities in its role as a putative CI accessory protein. Transcriptional profiling of *goa1∆* revealed a down regulation of genes encoding β-oligomannosyl transferases. Herein, we present data on cell wall phenotypes of *goa1*∆ (strain GOA31). We used transmission electron microscopy (TEM), GPC/MALLS, and NMR to compare GOA31 to a gene-reconstituted strain (GOA32) and parental cells. We note by TEM a reduction in outer wall fibrils, increased inner wall transparency, and the loss of a defined wall layer close to the plasma membrane. GPC-MALLS revealed a reduction in high and intermediate Mw mannan by 85% in GOA31. A reduction of β-mannosyl but not α-mannosyl linkages was noted in GOA31 cells. β-(1,6)-linked glucan side chains were branched about twice as often but were shorter in length for GOA31. We conclude that mitochondrial CI energy production is highly integrated with cell wall formation. Our data also suggest that not all cell wall biosynthetic processes are dependent upon Goa1p even though it provides high levels of ATP to cells. The availability of both broadly conserved and fungal-specific mutants lacking CI subunit proteins should be useful in assessing functions of fungal-specific functions subunit proteins.

## Introduction

The cell wall of *C*. *albicans* is a dynamic structure that provides critical cell functions such as protection from the hostile environment of the host, adherence to host surfaces, and maintenance of cell shape [1]. The dynamic status of the cell wall is reflected by its fluctuations in polysaccharide content during changes in growth forms (yeast and pseudohyphae/hyphae) as well as upon exposure to cell wall perturbing antifungal compounds [1]. The *C*. *albicans* cell wall is mainly composed of polysaccharides such as β-glucans, chitin, and mannan (Man) [2]. The proportion of each polysaccharide polymer in the cell wall of this organism is 47–50% β-glucan, ~40% mannan and 0.6–9% chitin [2–6]. Chitin and β-glucans constitute the inner cell wall while mannan/mannoproteins are primarily located at the cell surface. In comparison to model yeast (*Saccharomyces cerevisiae*), the cell wall of *Candida* species has expanded families of cell surface adhesins such as those of the ALS family (agglutinin-like sequences) of *C*. *albicans* and the epithelial adhesins (EPAs) of *C*. *glabrata*. *C*. *albicans* also has a greater amount of β-1,6 glucan. The outer mannan polysaccharides have both α-, β-, and phosphomannan linkages while *S*. *cerevisiae* lacks β-mannan. Each of these wall differences may be related to its survival as a commensal and a pathogen.

Mannan polysaccharides have α-1,6, α-1,2, α-1,3 and β-1,2-linkages in *C*. *albicans* [2]. β-linked galactomannans (β-1,5-linked oligosaccharide side chains) have also been identified in other fungal pathogens such as *A*. *fumigatus* [7]. Both types of α- and β-linked phosphomannan are required for innate immunity. In fact, attempts to develop a glycoconjugate vaccine against candidiasis were mostly designed as synthetic β-linked mannan (β-Man) [8–9]. The protective effect of monoclonal antibodies to β-linked mannans in animal models has been reviewed [10].

Two types of immunodominant mannans are localized to the cell surface, phosphopeptidomannan (PPM) and phospholipomannan (PLM) [4]. Most mannan chains in fungi are covalently linked to protein by *N*-glycosidic bonds to asparagine residues, accounting for 85% of mannans. The remaining proportion (15%) of PPMs is linked by *O*-glycosidic, alkali-labile linkages to serine or threonine residues. In contrast to the longer and more highly branched chains with phosphodiester bonds in *N*-linked mannoprotein, the *O*-linked mannoprotein usually contains a shorter, unbranched mannan [2]. The second type of immunogenic mannan is PLM, which is attached to cell membrane sphingolipids [11]. As with the PPM, serotypes of *Candida* spp. also depend upon the degree of mannan polymerization (mainly β-Man) in PLMs (11). Apparently, the polysaccharide chains, based on different combinations of β-Man and α-Man in PPM and PLM, provide spatial and temporal support for their immunogenic roles [2]. As stated above, pattern recognition receptors (PRRs) of immune cells such as macrophages, neutrophils, and dendritic cells bind these fungal pathogen-associated molecular patterns (PAMPs) and induce adaptive immunity [12–13].

The correlation between mitochondrial function and pathogenicity of *C*. *albicans* was first noted in our published studies of the mitochondrial mutant *goa1*Δ (GOA31) [14–17]. We found that Goa1p is required for mitochondrial complex I (CI) activity, alternate carbon metabolism, cell growth, and aging. Deletion of *GOA1* results in avirulence in a blood-borne, invasive candidiasis murine model [15]. Recognition by macrophages, epithelial cells, and phagocytosis by neutrophils is impaired in the *goa1*Δ mutant, causing a reduction in macrophage cytokine production [17]. However, non-phagocytized *goa1∆* cells are killed along with phagocytized organisms [15,17].

Cell energetics should be critical to cell wall biosynthesis and assembly. Mitochondria are known to associate with the Spitzenkörper structure, a cluster of vesicles that carry enzymes and wall precursors to the active growing tips of hyphae in *Neurospora crassa*, *Candida albicans*, and other fungi [18]. The coupling of ATP synthesis to cell wall polysaccharide biosynthesis in fungi must be synchronized through signal pathways such as the *S*. *cerevisiae* AMP-activated protein kinase pathway (AMPK), which monitors the intracellular ATP/ADP ratio and apparently links energy production with wall synthesis [19–22]. The regulation of these events is minimally studied in fungal pathogens. During infection, pathogens must adapt to conditions of low carbon (glucose) availability. Metabolic processes such as β-oxidation of stored lipid and gluconeogenesis are integrated as survival tactics [23]. The term “flexible metabolism” of *C*. *albicans* has been coined to illustrate pathways which conserve carbon, such as the peroxisomal glyoxylate cycle in *C*. *albicans*, assuring survival in the host [24–25]. In this regard, we hypothesize that a similar mechanism may contribute to the coordinated efforts of cell wall synthesis, metabolism and cell energetics in pathogenic *C*. *albicans* [17, 26–27]. To gain insight into mitochondrial functions in cell wall biosynthesis, we compared the loss of energy production in the GOA31mutant with changes in cell wall composition. Our current findings correlate with published data on transcriptional profiling in the same mutant [27]. The wall changes that occur in this mutant appear specific to *N*-glycosylated mannoprotein synthesis, especially β-linked mannosylation and a shortening but increase in the number of branching β-1,6 glucan chains.

## Materials and Methods

### Fungal strains, media, and culture conditions

Wild type SC5314 (WT) was used in all experiments. The *goa1*Δ mutant (GOA31) and its gene reconstituted strain (GOA32) have been described previously [14–17]. Fungal cells were routinely cultured in yeast extract peptone dextrose (YPD) broth containing 2% glucose at 30°C [15].

### Transmission electron microscopy (TEM)

We followed the procedure described previously by Hall *et al* [28]. Yeast cells were collected from exponential growth cultures as follows: 10^6^ cells/ml of WT, GOA31 and GOA32 were inoculated respectively into 10-ml of pre-warmed YPD medium and grown for 6 h (WT, GOA32) or 8 h for GOA31 at 37°C. Cells were immediately frozen in liquid nitrogen under high pressure using a Leica EM PACT2 (Leica Microsystems) and transferred by Leica EM AFS2. Prior to TEM analysis, samples were sequentially warmed to −30°C in acetone/OsO_4_ for 8h, then to 20°C for 3 h in acetone, and embedded in increasing amounts of Spurr (epoxy) resin for 24 h. Ultra-thin sections (100 nm) were stained with uranyl acetate and lead citrate and imaged with a Philips CM10 transmission electron microscope (FEI Life Sciences, Hillsboro, Oregon). To better evaluate the length of the cell wall and outer wall fibrils, 10 cells of each strain were randomly selected and 10 measurements from different sections of each cell were obtained using the GIMP version 2.8. Data are presented as the mean ± standard deviation of 100 measurements. Data were analyzed by One-Way ANOVA followed by Bonferroni’s test with a 99% confidence level and t-test using GraphPad Prism version 5.01.

### Total cell and cell wall hexose determinations

We used the phenol-sulfuric acid method as described previously to measure hexose content [29]. Each strain was cultured in 100-ml of M199 medium (pH 7.0) at 30°C for 6–9 generations before cells were collected. For dry weight determinations, quadruplicate cell samples were collected on pre-weighed nitrocellulose filters (pore size, 0.45 mm) and dried in vacuum. For cell wall hexose analyses, triplicate cultures of each strain were pelleted by centrifugation at 3,800 × g for 10 min, washed with ice-cold, sterile distilled water, and stored at -20°C prior to use.

Cell pellets were broken with glass beads and the crude cell wall of each strain was collected by centrifugation at 11,000 × g for 15 min and processed as described previously [29,30]. Cell wall extraction was done with an equal dry weight of all strains. Cell wall of each strain was fractionated as follows: for alkali extraction, cell wall was treated three times with 0.5 ml of 0.75 M NaOH for 60 min at 75°C. The three extractions were combined and neutralized with glacial acetic acid (alkali-soluble). The alkali-insoluble material was washed first with 100 mM Tris (pH 7.5), followed by a second wash with 10 mM Tris (pH 7.5), and suspended in the same buffer containing 0.01% sodium azide. Further extraction was done by adding 1 mg of β-1,3-glucanase (Zymolyase 20T, ICN Pharmaceuticals) followed by incubation for 16 h (37°C with gentle shaking). The supernatants are referred to as the Zymolyase-soluble fraction. The Zymolyase-insoluble material was collected by centrifugation (11,000 × g, 15 min) and washed with sterile, deionized water. The relative hexose content from each fraction was determined using a standard curve generated from different glucose concentrations [29,30]. From the Zymolyase-insoluble fraction, chitin determinations were made using a glucosamine standard. Data are expressed as μg hexose or hexosamine per mg of cell dry weight (mean ± standard deviation).

### Alcian blue binding by strains

We used methods described previously [31–32]. A standard curve of Alcian Blue was established using concentrations of 2–50 μg/ml. Exponential-phase yeast cells (1 × 10^7^/ml) of each strain (OD_600_ = 0.5) were harvested by centrifugation at 3,800 × g for 10 min at room temperature, washed with 1-ml of 0.02N HCl, and stained with 50 μg/ml of Alcian Blue in 1-ml of 0.02N HCl for 10 min at room temperature. Cells were pelleted and suspended in 1-ml of 0.02N HCl. To measure binding, the absorbance (OD_600_) of 100-μl of cell suspension was measured using an Ultrospec 2100 spectrophotometer (Amersham Sciences).

### β-Glucan isolation

Glucan was isolated from all strains using a modification of methods described previously [33]. Cell walls were extracted with 0.1 N NaOH for 15 min at 100°C followed by neutralization to pH 7.0 with 2M acetic acid. The precipitated residue was sequentially extracted with 0.1 N H_3_PO_4_ for 15 min at 100°C and neutralized again. The majority of the lipids were removed following treatment with 3-volumes of 100% ethanol at 100°C for 15 min. The water insoluble, microparticulate cell wall material was harvested by centrifugation (100,000 × g for 20 min), washed in ultrapure water several times, frozen, and lyophilized.

### Mannan extraction

We used our published procedure [31]. In brief, cells were harvested by centrifugation at 3,800 × g for 15 min from 2-liters of overnight cultures grown at 30°C in YPD medium. Cells were washed and dehydrated with acetone. The cell slurry was obtained after agitating the cells in 100-ml of water with glass beads followed by autoclaving for 3 h. Cell debris was removed, and the supernatants of each sample were collected. An equal volume of freshly prepared Fehling’s solution was mixed with the supernatant fractions for 1 h [34,35]. The resulting precipitate was then harvested and treated with 3N HCl to dissolve the copper-mannan complex. The soluble copper-mannan solution was then mixed with 50-ml of methanol:acetic acid (8:1), and the precipitate was allowed to settle overnight and washed with methanol several times. All supernatants were decanted. The material was then air-dried, suspended in dH_2_O, and the pH was adjusted to 6.5–7.0. Using this approach, we generally recovered 100–250 mg of mannan from 10–15 g of cell wet weight from the wild type strain.

### Gel permeation/multi-angle laser light scattering (GPC/MALLS) analysis of mannan

Duplicate mannan samples extracted from each strain were used. Mannans were diluted to 3 mg/ml in the mobile phase (50 mM NaNO_2_) at ambient temperature for 15 min, or heated at 60–80°C for 30 min to improve the solubility. The samples were then filter-sterilized (0.22 μm) into 1-ml injection vials and analyzed over a concentration of 300–600 μg in 100–200 μl. We used a Viscote GPCMax autosampler and pump with the flow maintained at 0.5 ml/min. Three Ultrahydrogel GPC columns (1200, 500,100) were connected in a series [34]. Prior to sample analysis, the GPC system was validated with water-soluble pullulan (Viscotek and Showa Denko) and dextran standards (1 × 10^5^ to 1.66 × 10^6^ g/Mol). The dn/dc (Index of Refraction) for mannan samples was calculated to be 0.14. The inter- and intra-experimental variability was ≤ 3 ± 1%, depending upon the standard employed. Data were acquired with TDA 305 (Viscotek) multi-detector system and analyzed with Viscotek OmniSec (ver 4.7.0.406) software.

Proton NMR spectra for mannan [36] and glucan [37] were collected on a Bruker Avance III 600 NMR spectrometer using a CH cryoprobe operating at 345°K (72°C) in 5-mm NMR tubes as reported previously. Mannan (about 10 mg) was dissolved in about 600 μl D2O (Cambridge Isotope Laboratories, 99.8+% deuterated). Chemical shift referencing was accomplished relative to TMSP at 0.0 ppm (parts per million). NMR spectra were collected and processed as follows: 16 scans, 32,768 data points, 25 ppm sweep width centered at 5.0 ppm, 15 s pulse delay, and exponential apodization with 0.2 Hz broadening. Mannan NMR spectra were processed using wxMacNUTS (2nd Generation NMR Utility Transform Software, Version 1.0.1, Acorn NMR, Inc.) on a Macintosh MacBook Pro. Spectral comparisons in pairs were used to detect structural changes as indicated by changes in assigned peak intensities. For each set of comparisons, the spectra were height normalized to the largest peak in each spectrum. The resonance in each spectrum at 5.08 ppm was assigned to the anomeric proton of α-(1–6)-linked mannosyl repeat units in the backbone with α-(1–2)-linked mannosyl repeat units attached.

About 10 mg of the glucan was dissolved in 1 ml of DMSO-d6 at 80°C. A few drops of trifluoroacetic acid-d (99.8% deuterated, Cambridge Isotope Laboratories) were added to the solution to shift the exchangeable proton resonance downfield. NMR chemical shifts were referenced to the residual DMSO-d6 proton resonance at 2.50 ppm. The NMR spectral collection and processing parameters were the following: 16 scans, 32,768 data points, 25 ppm sweep width centered at 5.0 ppm, 15 s pulse delay, and exponential apodization with 0.2 Hz broadening. Glucan NMR spectra were processed using JEOL DELTA software version 5.0.3 on a Macintosh MacBook Pro. Branching frequency and side chain length were calculated as reported previously [38].

### Statistical analyses

TEM and Alcian blue data were analyzed by One-way ANOVA followed by the Bonferroni’s test with a 99% confidence level using GraphPad Prism version 5.01. Data obtained from cell wall hexose analysis and Alcian blue binding were determined from three independent biological samples and analyzed by the two-way ANOVA test following Bonferroni posttest with a 95% confidence level using GraphPad Prism version 5.01.

## Results

### Ultrastructural changes occur in the GOA31outer cell wall

**Fig 1:**
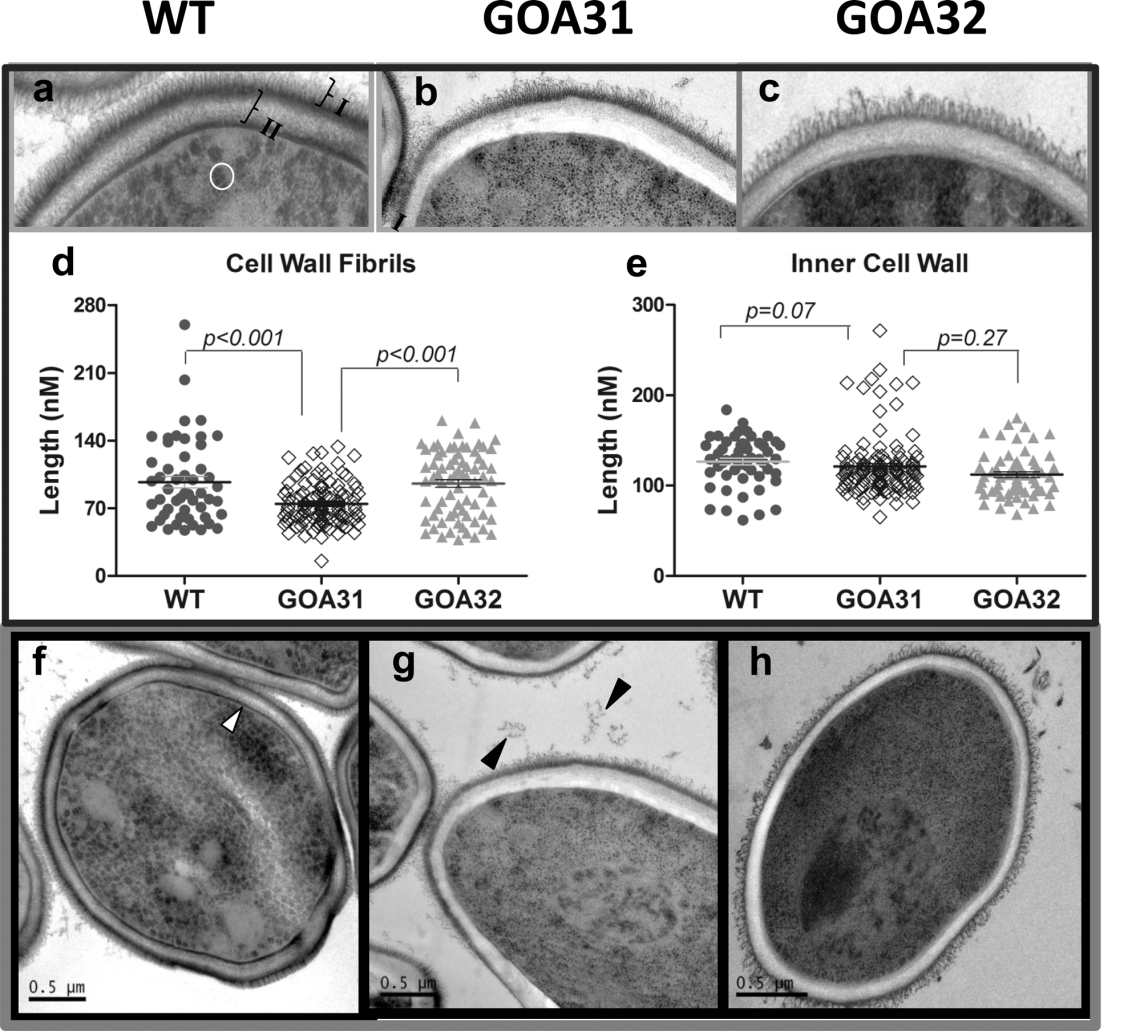
GOA31 displays ultrastructural changes in the outer and inner cell wall. TEM of *C*. *albicans* **strains**: **a,f** = WT; **b,g** = GOA31; **c,h** = GOA32. Differences are noted in the amount and length of the cell surface fibrils with GOA31 compared to the WT cells (“I”, **Fig1a**) and GOA32. Also, the inner cell wall (“II”, **Fig 1a**) is more electron dense than that in GOA31, as well as the cell wall layer closest to plasma membrane (white arrow, **Fig1f**). Both are reduced only in GOA31. The average length of cell wall fibrils (**d**) and **inner** cell wall **(e)** are also determined. The fibrils are significantly shorter in GOA31 but the cell wall thickness is similar for all strains. In (**d,e**) each data point refers to an collection of 10 measurements of 10 cells for each strain using GIMP2.8 software. Vesicles (circle) in WT (**Fig1a)** are significantly reduced in GOA31. The black arrows may indicate sloughed cell wall material in **Fig 1g**.

We analyzed the cell wall ultrastructure of all strains using TEM and noted major differences in GOA31 compared to control strains (Fig 1). WT cell wall (Fig 1A and 1F) exhibits electron-dense layers of outer fibrils (I, Fig 1A), inner wall (II, Fig 1A), and a typical layer in juxtaposition to the cell plasma membrane (white arrows, Fig 1F). The cytoplasm of WT cells contains numerous, dark, circular vesicles (white circle, Fig 1A). The cell wall of GOA32 (reconstituted with a single *GOA1* allele) is similar in architecture to WT cells but with some reduction in electron density (Fig 1C and 1H). In comparison, the GOA31 cell wall had three major morphological changes as observed by TEM (Fig 1B and 1G). The outer cell wall surface is less fibrillar, and fibrils are shorter than ones of WT cells (Fig 1D, *p< 0*.*01*). The second change is that the inner cell wall is more electron-transparent although the cell wall diameter is similar to control strains (Fig 1E, p = 0.07). Third, there is no clear demarcation of an inner membrane-associated wall layer (Fig 1B and 1G). The outer fibrillar wall is the location of *N*-linked mannan that is a component of the adherence glycoproteins [2]. GOA31 had fibrillar material external to cells, black arrows (Fig 1G). The reduction in cell surface fibrils correlates with transcriptional data in the GOA31 mutant, in which the ALS gene family of adherence glycoproteins as well as genes encoding β-linked mannan are down regulated [27].

### GOA31 has reduced cell and cell wall hexose content

**Fig 2:**
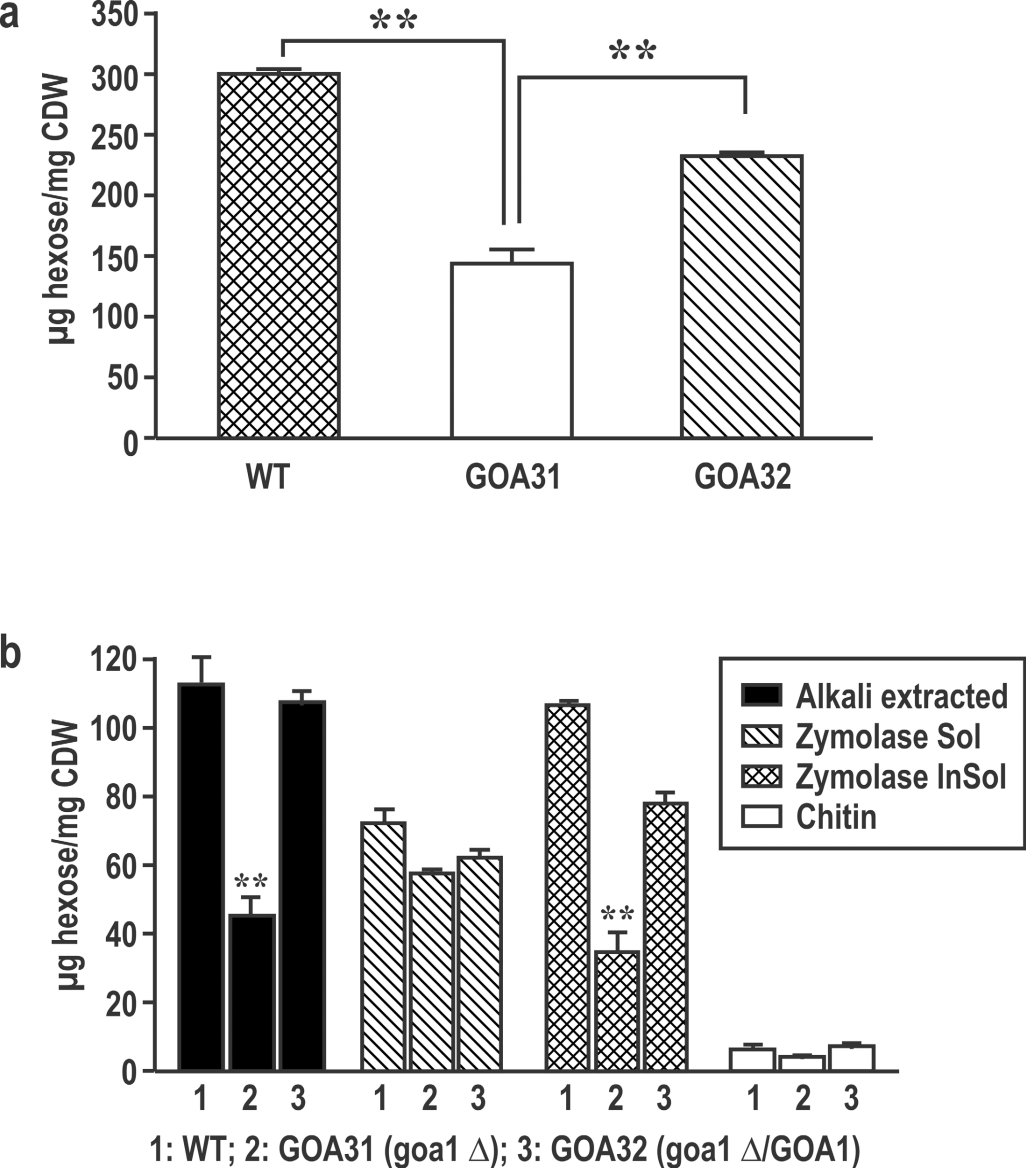
Reduced cell and cell wall hexose content of GOA31. Whole cell (**a**) and cell wall fractions **(b)** from strains of *C*.*albicans*, WT, GOA31 and GOA32. Total cell hexose content of GOA31 is significantly reduced compared to control strains. In **(b),** the hexose content of alkali-soluble, Zymolyase-soluble, Zymolyase-insoluble, and chitin content are shown. In GOA31, the hexose content is lower in the alkali soluble and Zymolyase-insoluble hexose compared to WT and GOA32, but Zymolyase-soluble and chitin fractions were similar in all strains.

All strains were analyzed for total cell and cell wall hexose, the latter from fractions that are designated as alkali-soluble, Zymolyase-soluble, Zymolyase-insoluble, and chitin (Fig 2). Total cell hexose is reduced in GOA31 compared to WT and GOA32 (*p<0*.*00*1) (Fig 2A). In GOA31, a significant reduction in hexose is observed in the cell wall alkali-soluble and Zymolyase-insoluble fractions compared to WT and GOA32 (*p< 0*.*001*) but not in Zymolyase-soluble and chitin content (Fig 2B). The similar levels of hexose in the zymolyase-soluble and chitin fractions suggest that β-1,3 glucan and chitin are less affected in GOA31.

### *C*. *albicans* GOA31 binds less Alcian blue

**Fig 3:**
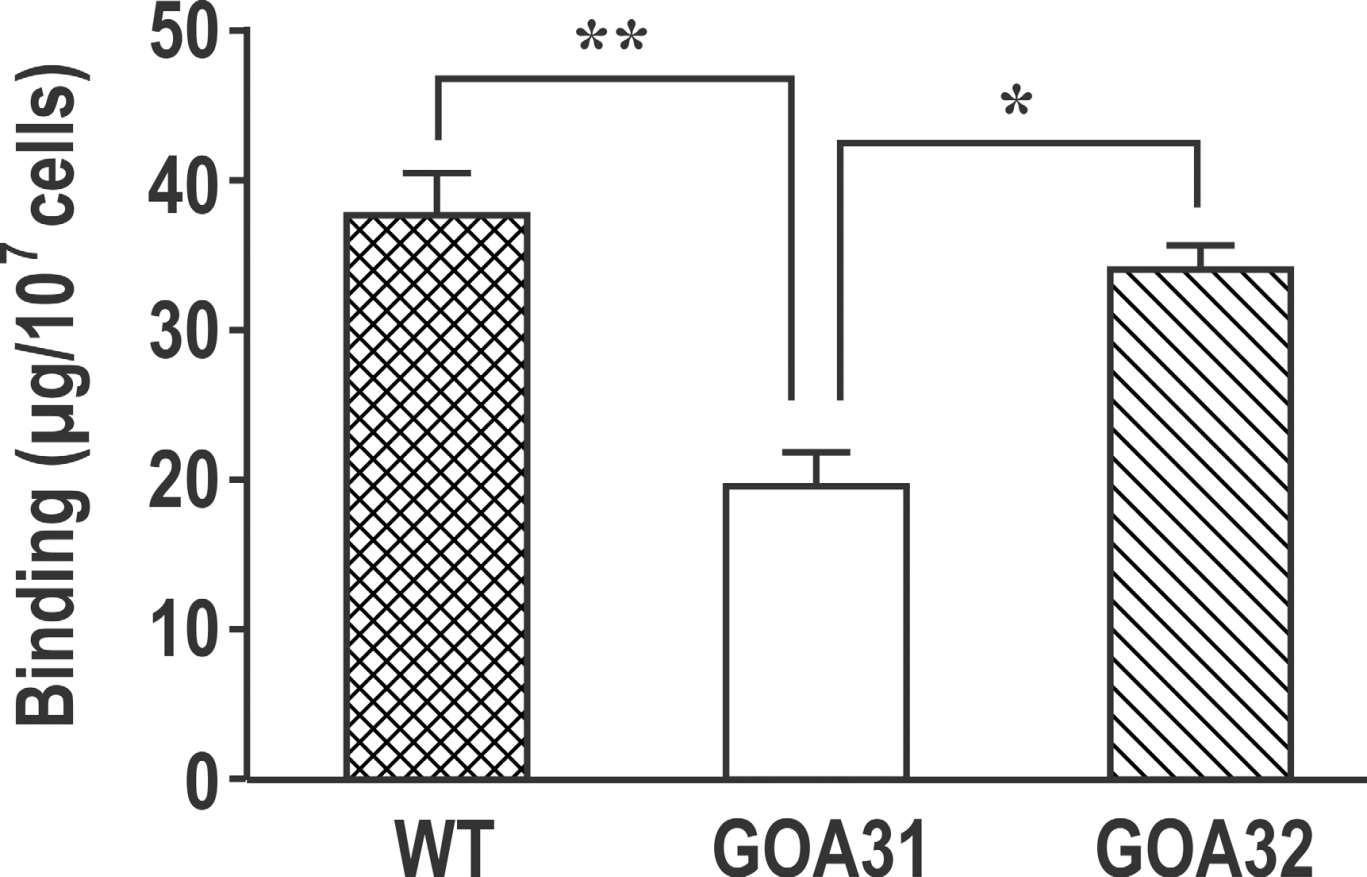
Alcian blue staining of whole cells is reduced in GOA31. Strains WT, GOA31, and GOA32 are compared. Binding by GOA31 is significantly reduced compared to other strains (p<0.005).

Phosphomannan side chains in *N*-linked mannan/mannoprotein and phospholipomannan (PLM) are both important immunogenic moieties for host-pathogen interactions. The mannosylphosphate residues in both PPM and PLM confer a net negative charge to the cell wall. Alcian blue is a cationic dye and consequently binds to the negatively charged phosphomannan cell surface [17]. Compared to WT cells, Alcian blue binding was reduced by ~50% in GOA31 (*p*<0.005) (Fig 3), while binding of Alcian blue by GOA32 was similar to WT cells. These data suggest a reduction in GOA31 phosphomannan.

### High and intermediate molecular weight mannan are absent in GOA31

**Fig 4:**
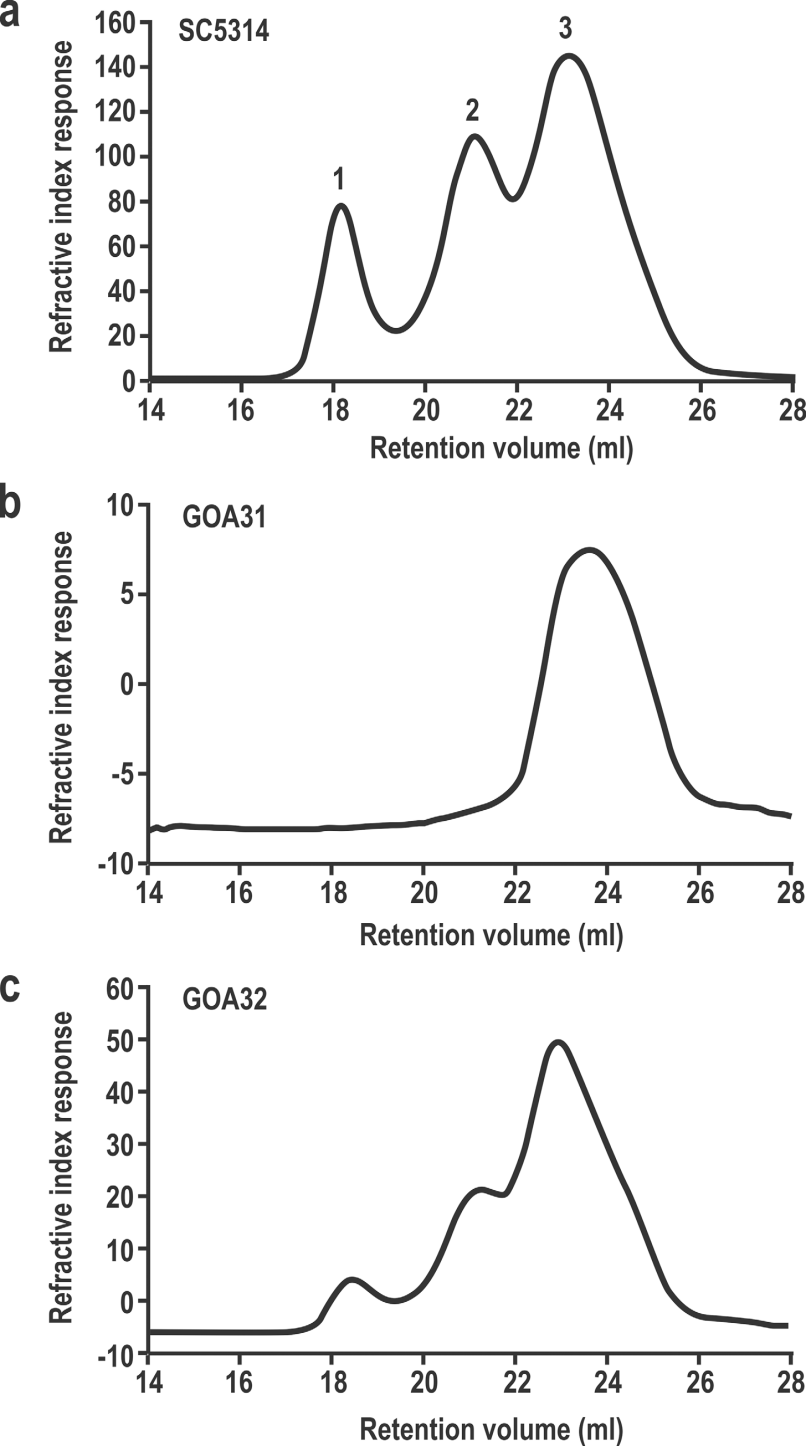
High and intermediate mannan classes are absent in GOA31. GPC-MALLS analyses of mannan reveals three molecular sizes (1–3) from WT (**Fig 4a**) and GOA32 cells (**Fig 4c**). In GOA31 (**Fig 4b**), both high- (1) and intermediate- (2) sized mannan fractions are not apparent.

To explain the reduction of wall hexose content, we analyzed mannan from GOA31 using GPC-MALLS chromatography (Fig 4). WT cells yielded a typical trimodal polymer distribution of which the molecular weights (Mw) for individual mannan/mannoprotein peaks are 38.4, 6.2, and 1.2 × 10^5^ g/Mol, corresponding to high, intermediate and low Mw polysaccharides (Fig 4A). GOA31 lacked both high and intermediate polysaccharide fractions (Fig 4B), while GOA32 also showed a trimodal polymer distribution although the overall Mw(s) for each peak were slightly altered compared to WT cells. We found that the Mw (s) in three peaks of GOA32 (Fig 4C) were 31.5 ×, 6.3 × and 1.4 × 10^5^ g/Mol. Other physiochemical data from the three strains are described in **Table 1**. The average Mw(s) from GOA31 and GOA32 were 85.1% and 48.9% lower than the WT mannan (9.4 × 10^5^ g/Mol), respectively. These changes are primarily due to loss or significant attenuation of the two larger Mw peaks mentioned above. In addition, the polydispersity, defined as a measure of the distribution of molecular mass in a given polymer sample, the Mark-Houwink (α values), and the hydrodynamic volume (Rh, volume of polysaccharide in solution) are clearly different in GOA31 compared to control strains (**Table 1**). The physicochemical descriptions for GOA32 are much more comparable to WT. Perhaps, the remarkable loss of hexose in the GOA31 alkali-soluble portion (Fig 2B) is due to a reduction in mannan and β-1,6- glucan changes observed by GPC-MALLS as described below.

**Table 1:** GPC-MALLS analysis of cell wall mannoprotein/mannans in *C*. *albicans* mutant.

Sample	Mw^a^ (x 10^5^ g/Mol)	% Δ^e^	Polydispersity^b^ (Mw/Mn)	% Δ^e^	Mark-Houwink^c^ (α)	% Δ^e^	Rh^d^ (nm)	% Δ^e^
**SC5314**	9.4	---	4.8	---	0.57	---	14.4	----
**GOA31**	1.4	-85.1	1.8	-62.5	0.39	-31.6	8.0	-44.4
**GOA32**	4.8	-48.9	3.4	-29.2	0.52	-8.8	12.8	-11.1

^a^ average molecular weight of the mannoprotein/mannan samples expressed as g/Mol. Each sample was analyzed induplicate or triplicate.

^b^ polydispersity is a measure of the distribution of molecular mass in a given polymer sample. Polydispersity is calculated as the weight average molecular weight (Mw) divided by the number average molecular weight (Mn).

^c^ the slope of the liner relationship between log intrinsic viscosity and log molecular mass ([η] = *K*_a_M^a^) is the Mark-Houwink or α-value for a polymer system. The α value can provide insights into the solution conformation of the polymer system.

^d^ Rh = hydrodynamic radius. Rh (presented in nm) describes the volume of a polymer when it is in solution. Rh depends upon the nature of the polymer, the solvent and the temperature. In this analysis solvent and temperature were controlled, thus allowing examination of polymer nature.

^e^ percent change compared to wild type SC5314

### NMR spectroscopy reveals a decrease in GOA31 β-1,2 linked mannan linkages

**Fig 5:**
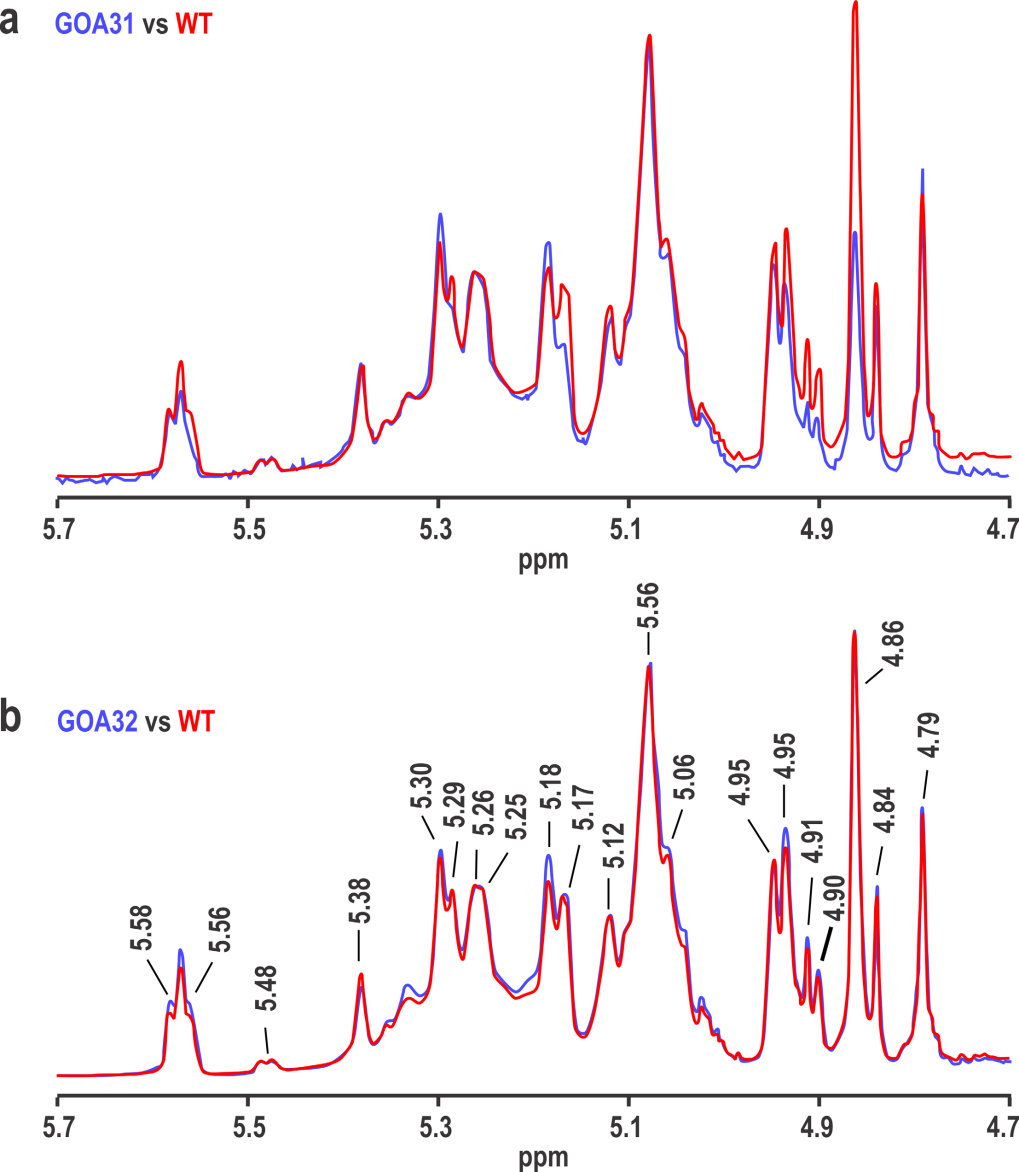
NMR reveals a reduction in β-1,2 linked mannan (peak 4.86) of GOA31. The 600 MHz proton NMR spectra of mutants (in blue) GOA31 (top) and GOA32 (bottom) are compared to the NMR spectrum of WT (in red) by overlaying the spectra and adjusting the resonance at 5.08 ppm to the same height in each pair of spectra. A prominent loss of the 4.86 ppm resonance (β-1,2 mannan) occurred in GOA32.

The inability to maintain the higher and intermediate molecular weight mannan suggests that either less synthesis or instability may occur in GOA31. To answer the former, the NMR spectra of GOA31 (Fig 5A) (blue) and GOA32 (Fig 5B) (blue) are compared to that of WT mannan (red). In the low molecular weight mannan, the overall structure of GOA31 is similar to that of WT except for some changes in the acid-labile portion of mannan. Data show a slight loss in intensity of resonances at 5.56, 4.91, and 4.90 ppm, but a significant loss at 4.86 ppm in GOA31 (Fig 5A). On the other hand, all resonances even at 4.86 ppm in GOA32 NMR spectrum are similar to WT (Fig 5B). These data indicate that all α-linkages of GOA31 are similar to WT cells and support transcriptional profiles (27).

### Goa1p is required for glucan branching but not biosynthesis

The inner skeletal wall contains linear and branching β-1,3 glucan as well as chitin while the outer layer contains β-1,6 glucan and cell wall glycoproteins that are attached to the skeletal wall by glycosylphosphatidylinositol (GPI) anchors (28). Most cell wall glycoproteins (CWPs), such as those of the ALS family, are GPI-anchored and linked through highly branched β-1,6 glucan chains to β-1,3 glucan.

In the present NMR study of β-glucan, the average side chain length (SC Length) and branching frequency (BrFreq) of the WT are 6.6 repeat units and 12.4 repeat units, respectively as described in **Table 2**. For GOA31 and GOA32, side chains occur about twice as frequently as in WT cells but are only about half the length of WT in GOA31. Comparing the level of side chain repeat units versus the backbone repeat units (SC/BB), GOA31 and GOA32 contain roughly equal side chain levels at 65 and 64%, respectively, while WT has about an 18% lower side chain content compared to GOA31 and GOA32. These data suggest that the β-1,6 linked glucan side chain in GOA31 is shorter but more frequent. However, a gene dosage affect is not significant in GOA32 in terms of SC and Br Freq parameters.

**Table 2:** *NMR analysis of glucans from C*. *albicans strains*.

Sample	SC length^a^	% Δ^b^	Br Freq^c^	Br fold^d^	SC/BB^e^
**SC5314**	6.6	---	12.4	---	53%
**GOA31**	3.6	-46.0	5.5	2.3	65%
**GOA32**	4.1	-38.0	6.4	1.9	64%

^a^ the average β-(1,6)-linked glucosyl side chain (SC) length.

^b^ percent change compared to wild type SC5314.

^c^ branching frequency

^d^ side chains of GOA31 and GOA32 occur more frequently than WT cells

^e^ the level of side chain repeat units versus the backbone repeat units

## Discussion

Our previous studies focused on the phenotypes of *C*. *albicans* mitochondrial null mutants lacking the Complex I (CI) electron transport chain (ETC) genes (*NUO1*, *NUO2*, *NDH51*) and a putative regulator of CI (*GOA1*) [14–17, 26–27]. Transcriptional profiling of these mutants revealed some functional overlap but also significant differences [27]. Nuo1p and Nuo2p are not found in mammalian cells. Their similar transcription profiles suggest functionally redundant proteins. Ndh51p is a broadly conserved CI subunit protein, while, as mentioned above, Goa1p may be a CI accessory protein that regulates NADH-oxidoreductase activity. For this manuscript, we chose to focus upon Goa1p and its role in cell wall construction, while comparing transcription profiles to cell wall biochemical and structural data in the null strain GOA31.

A link between mitochondrial metabolic activity and cell wall composition has been described briefly in *S*. *cerevisiae* [39]. Phosphopeptidomannans were less stable in mitochondrion-deficient cells than in wild type cells grown aerobically; a consequence of deficient mitochondria was a decreased cell flocculation [39]. In *C*. *albicans* GOA31 flocculation is increased [17]. Starvation in *S*. *cerevisiae* resulted in a disappearance of cell surface mannan that could be restored when cells were returned to favorable growth conditions. Since *S*. *cerevisiae* lacks a CI and β-linked mannan, comparisons to *C*. *albicans* are somewhat limited.

The roles of the *C*. *albicans* cell wall in host responses, growth, pathogenesis, and virulence are well known [40,41]. During invasion, a growth switch from yeast to filamentous hyphae occurs that is accompanied by cell wall changes. For example, β-1,3 glucan biosynthesis is more active during early germ tube formation [41,42]. When grown in glucose-rich medium, *C*. *albicans* appears to build a remarkably thick cell wall, which was reduced by 50% in lactate-medium. However, the cells grown in lactate gained resistance to osmotic stress, antifungal agents and cell adherence [43]. Under carbon limitation, the different responses between non-pathogenic *S*. *cerevisiae* and pathogenic *C*. *albicans* in regard to cell wall composition and mannoproteins may suggest an adaptation to the host environment in the pathogen.

Images of the *C*. *albicans* cell wall reveal a layered structure that is associated with the distribution of polysaccharides such as chitin, the β-glucans and mannoproteins, the latter of which is dispersed within a network of structural glucan and chitin polymers [4]. Mannoproteins dominate the outermost cell wall layer and form fibrillar extensions. β-1,6 glucan has a central role in the organization of the yeast cell wall since it interconnects the inner wall of β-1,3 –glucan and chitin with the outer layer mannoprotein [44].

Synthesis of cell wall components requires energy, most of which is provided through oxidative phosphorylation by the mitochondrial ETC. Our interest was to determine what changes in wall construction occur during energy (ATP) limitations. A secondary aim was to correlate transcriptional changes [17] with cell wall biochemical modifications. The published data show that GOA31 was unable to oxidatively phosphorylate ADP and had an 80% reduction in NADH-oxidoreductase activity [15,16]. Our initial expectation was that wall synthesis would be affected broadly to reflect the severe loss in energy synthesis. But interestingly, the processes affected were primarily restricted to wall mannan and a shortening but increase in the number of branching β-1,6 glucan chains in GOA31. In the NMR study, a defect in β-1,2 mannan linkage is noted in the long phosphate side chains of *N*-linked mannan based upon change in the intensity of a resonance at 4.86 ppm. Presumably, these long chains contain more than one β-(1–2)-linked mannosyl repeat unit such as β-Man-(1–2)-[β-Man-(1–2)-]n-β-Man-(1–2)-α-Man-(1-PO4. Meanwhile, other linkages (α-1,6 Man, α-1,2 Man, α-1,3 Man) were relatively unaffected.

These data are in fact consistent with the transcriptional data of GOA31 described previously [27]. The down-regulated genes included β-mannosyltransferases (*BMTs)*, phosphomannan and glucanases, but genes encoding for chitin, β-1,3 and β-1,6 glucan synthase genes were relatively unaffected. In the group of *BMTs*, *BMT3* reduction was most prominently down regulated. The gene product of *BMT3* is assigned to the second β-1,2 Man unit linkage. Down regulation was also noted for *BMT6* (β-1,2 Man extension in PLM) as were *BMT7-9*, but the precise roles of *BMT*7-9 in β-mannoside elongation have not yet been determined [45]. In addition, we found 4/5 genes that are responsible for the formation of mannosylphosphorylation linkages are also down-regulated [27], which may also contribute to the reduction of long phosphate acid-labile chains in NMR results.

GPC-MALLS data clearly demonstrated a great loss of high- and intermediate-sized mannan in GOA31. One could question why the small portion of β-mannan (5–10% in wild type) resulted in 85% mannan reduction in this mutant. Our results agree with the concept that the physical features of β- mannosyl repeating units are unique and provide an important spatial configuration for the mannan structure [44]. Unfortunately, the mechanisms and processes of β-Man polymerization are poorly understood. In general, the extension of polysaccharides at the vicinal C1 and C2 centers leads to medium-range steric clash between two residues; apparently β-mannosylated polysaccharide oligomers are less flexible than most other linkage types [6,46]. Rees and Scott have described β-1,2 mannan as crumpled, contoured chains [47]. They predicted that 1,2-linked sugars, such as β-1,2 mannan in *Candida*, either as a terminal or discontinuously as a component of a heteropolymer, can prevent the potential steric clash between non-adjacent sugar residues. According to these chemical features of β-mannan, we cannot exclude the possibility that shorter and fewer fibrils in GOA31 was caused by an instability of the remaining α-mannan chain. The instability may account for the release of extracellular wall material (see Fig 1G). However, this presumption needs to be verified by additional experiments.

Compared to WT cells, NMR analysis of the β-1,6 glucan side chain length is reduced by about 46% in GOA31, but the frequency of such branching is increased. We did not observe significant changes of chitin and hexoses in the zymolyase-soluble fraction in this study, perhaps because of changes in β-1,6 glucan scaffolding that did not affect synthesis of chitin and hexose. Apparently, the transparent inner cell wall in GOA31 may be due to the defects in glucan branching. In turn, this change likely causes inadequate attachments of GPI-anchored glycoproteins such as the ALS gene family that are covalently linked to β-1,6-glucan. Also, proteins with internal repeats (Pir1), may not be linked properly due to transcriptional down regulation of glucanases [27]. The latter are structural cell wall proteins and believed to crosslink β-1,3-glucan chains through ester linkages [48]. Intriguingly, in GOA31, *PIR1* and a significant number of GPI-synthases were down-regulated [27], for example, GPI-Gnt (acetylglucosaminyltransferase) that is required for the first step in GPI biosynthesis.

**Fig 6:**
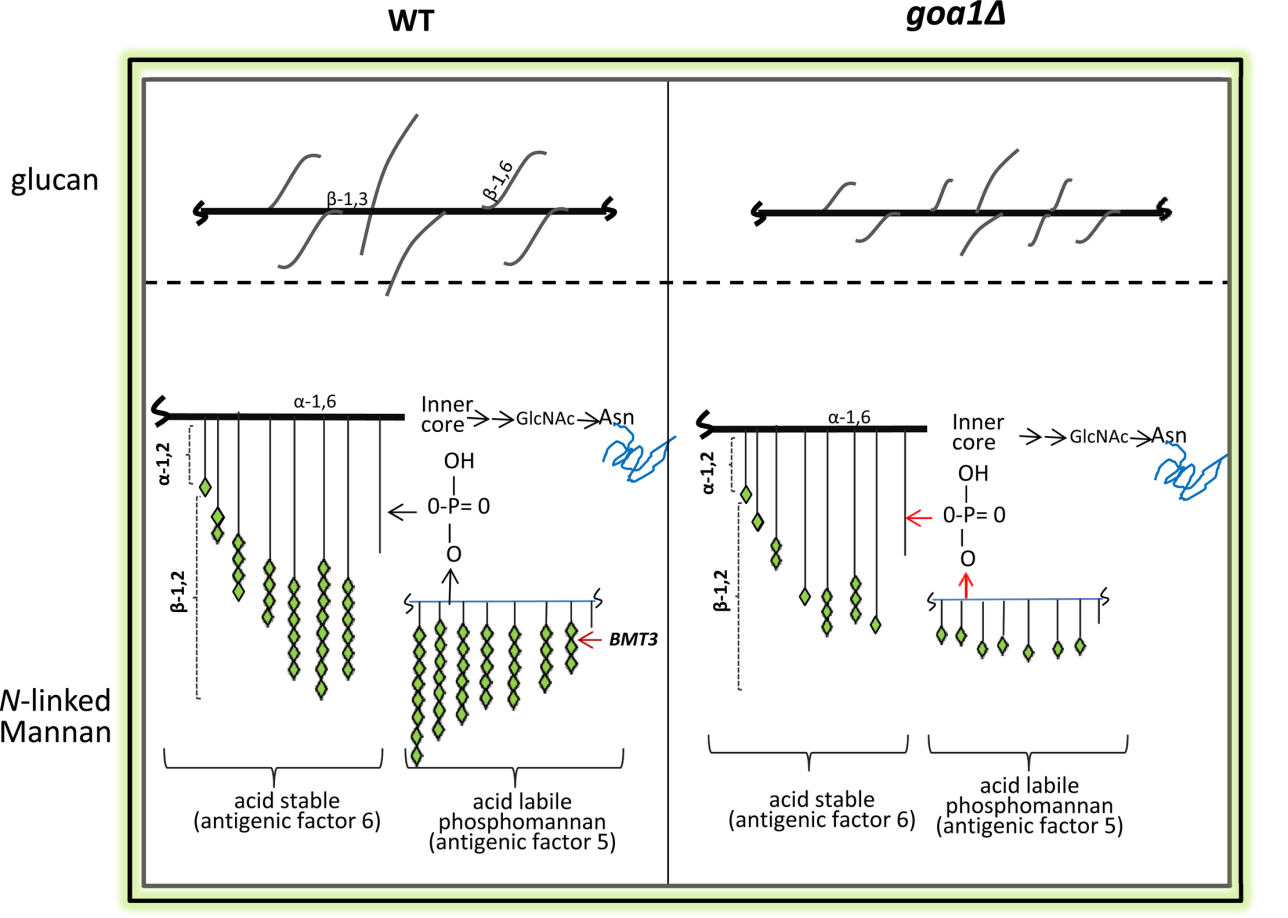
Models of the cell wall polysaccharides and linkages are shown for WT (left) and *goa1∆* (GOA31, right). Our data predict that GOA31 β-1,6 glucan is more highly branched but with shorter side chains compared to WT cells (upper). For *N*-linked mannan, β-linked mannans, particularly of acid-labile phosphomannan, are truncated in GOA31 compared to WT cells due to defects in β-1,2 mannan elongation.

In WT cells, β-mannosylated glycoconjugates elicit a strong antibody response and induce protection against mucosal colonization in mice [49,50]. As shown in Fig 6, β-mannosylation of sugar residues appears in both acid-stable and acid-labile portions of *N*-linked mannoprotein. Therefore, it is not surprising that cell wall changes in the mutant cause a reduced expression of macrophage TLR2 and TLR4 toll-like receptors [17]. TLR2 and TLR4 are thought to interact with phospholipomannan and *O*-linked mannan of PAMPs as well [13, 38]. Thus, PLM and *O*-linked mannan may also be responsible for decreased expression of PRRs.

At present, our model suggests that β-mannan, but less so α-mannan synthesis, is dependent upon Goa1p (**Fig 6**). These data suggest a degree of functional specificity for Goa1p and wall synthesis. We believe this is the first integrative study to suggest that the functions of a CI regulator are associated with key processes in cell wall synthesis. Because of their importance to *C*. *albicans* viability, we will continue to analyze the functions of the fungal-specific CI proteins Nuo1p and Nuo2p. We expect that their vital roles in cell energetics and fungal-specificity may warrant them as optimal targets for antifungal drug discovery.

## Abbreviations

ALS: agglutinin-like sequences
BMT: β-mannosyltransferase
CI: complex
CWP: cell wall protein
EPAs: epithelial adhesins
ETC: electron transport chain
GPC/MALLS: gel permeation chromatography/ multi-angle laser light scattering
GPI: glycosylphosphatidylinositol
GPI-Gnt: acetylglucosaminyltransferase
Man: mannan
NMR: nuclear magnetic resonance
PAMP: pathogen-associated molecular patterns
PIR: protein containing internal repeats
PLM: phospholipomannan
PPM: phosphopeptidomannan
ppm: parts per million
PRR: pattern recognition receptors
TEM: transmission electron microscopy
